# Digitale Gesundheitskompetenz von Schülerinnen und Schülern. Ausprägung und Assoziationen mit dem Bewegungs- und Ernährungsverhalten

**DOI:** 10.1007/s00103-022-03548-5

**Published:** 2022-06-02

**Authors:** Kevin Dadaczynski, Katharina Rathmann, Julia Schricker, Ludwig Bilz, Gorden Sudeck, Saskia M. Fischer, Oliver Janiczek, Eike Quilling

**Affiliations:** 1 Fachbereich Gesundheitswissenschaften, Hochschule Fulda, Leipziger Str. 123, 36037 Fulda, Deutschland; 2 Public Health Zentrum (PHZF), Hochschule Fulda, Fulda, Deutschland; 3 Zentrum für Angewandte Gesundheitswissenschaften, Leuphana Universität Lüneburg, Lüneburg, Deutschland; 4 Vestische Kinder- und Jugendklinik Datteln – Universität Witten/Herdecke, Datteln, Deutschland; 5 Fakultät für Soziale Arbeit, Gesundheit und Musik, Brandenburgische Technische Universität Cottbus-Senftenberg, Cottbus, Deutschland; 6 Institut für Sportwissenschaft, Eberhard Karls Universität Tübingen, Tübingen, Deutschland; 7 Interfakultäres Forschungsinstitut für Sport und körperliche Aktivität, Eberhard Karls Universität Tübingen, Tübingen, Deutschland; 8 Hessische Arbeitsgemeinschaft für Gesundheitsförderung e. V., Frankfurt am Main, Deutschland; 9 Department für Angewandte Gesundheitswissenschaften, Hochschule für Gesundheit, Bochum, Deutschland

**Keywords:** Gesundheitsinformationen, Digital Public Health, Jugendgesundheit, Obst- und Gemüsekonsum, Körperliche Aktivität, Health information, Digital public health, Adolescent health, Fruit and vegetable consumption, Physical activity

## Abstract

**Hintergrund:**

Während vermehrt Studienbefunde zur allgemeinen Gesundheitskompetenz (GK) vorliegen, mangelt es an Erkenntnissen zur digitalen GK im Jugendalter und deren Assoziationen mit Indikatoren des Gesundheitsverhaltens.

**Methodik:**

Empirische Basis bildet eine von Oktober 2019 bis Februar 2020 im Bundesland Hessen durchgeführte Querschnittstudie mit 490 Schülerinnen und Schülern der Klassenstufe 8 und 9. Die digitale GK wurde mithilfe von 5 Subskalen des Digital Health Literacy Instrument (DHLI) erfasst, während der Verzehr von Obst, Gemüse und Softdrinks sowie die körperliche Aktivität als Indikatoren des Gesundheitsverhaltens herangezogen wurden. Als soziales Merkmal wurde neben dem Geschlecht und der Klassenstufe der subjektive Sozialstatus (SSS) berücksichtigt. Die Datenauswertung erfolgte uni-, bi- und multivariat, wobei binärlogistische Regressionen für das Geschlecht und den SSS adjustiert wurden.

**Ergebnisse:**

Über alle Items hinweg berichten 15,3–37,5 % der befragten Jugendlichen Schwierigkeiten bei der Beschaffung von und im Umgang mit digitalen Informationen. Differenziert nach sozialen Merkmalen finden sich für 2 Dimensionen der digitalen GK Unterschiede zuungunsten der Mädchen sowie durchgehend ein sozialer Gradient zuungunsten von Befragten mit niedrigem SSS. Jugendliche mit mittlerer und geringer digitaler GK weisen ein höheres Maß an geringer körperlicher Aktivität, nichttäglichem Obstverzehr und täglichem Konsum von zuckerhaltigen Getränken auf.

**Diskussion:**

Die Befunde weisen auf einen Interventionsbedarf zur Förderung der digitalen GK insbesondere bei Jugendlichen mit geringem SSS hin. Die differenziellen Zusammenhangsmuster mit dem Gesundheitsverhalten bieten Ansatzpunkte für die Entwicklung spezifischer Interventionen. Als Lehr- und Lernort stellt die Schule u. a. aufgrund der Passung mit verpflichtenden Strategien der schulischen Medienkompetenzbildung ein geeignetes Setting dar.

## Abstract (en)

**Background:**

While there are increasing empirical findings on general health literacy (HL), there is a lack of evidence on digital HL in adolescence and its association with health behavior.

**Methods:**

A cross-sectional study of 490 students from grade eight and nine from the federal state of Hesse was conducted from October 2019 to February 2020. Digital HL was assessed using five subscales of the Digital Health Literacy Instrument (DHLI), while consumption of fruits, vegetables, and soft drinks as well as weekly physical activity were used as indicators of health behavior. In addition to gender and grade level, subjective social status (SSS) was used as a social characteristic. Univariate, bivariate, and multivariate analyses were performed, with binary-logistic regression adjusted for gender and SSS.

**Results:**

Across all items, 15.3 to 37.5% of adolescents reported difficulties in acquiring and dealing with digital health information. Stratified by social characteristics, gender and socioeconomic differences were found with girls and respondents reporting a lower SSS more often showed a limited digital HL. Adolescents with moderate and low digital HL reported higher levels of low physical activity and non-daily fruit and daily soft drink consumption.

**Discussion:**

The findings suggest a need for interventions to promote digital HL among adolescents, particularly for those of low SSS. In this context, the differential relationship patterns with health behaviors provide an avenue for the development of specific interventions. The school as a place of teaching and learning is a suitable setting because, among other things, of its fit with the mandatory strategy of media literacy education in schools.

## Einleitung

Auch wenn der Begriff „Digital Natives“ u. a. aufgrund seiner mangelnden empirischen Fundierung durchaus Gegenstand der kritischen Diskussion ist [1], dürfte unstrittig sein, dass junge Menschen in hohem Maß mit digitalen Medien und Informationsangeboten aufwachsen. Die Ergebnisse der vom Medienpädagogischen Forschungsverbund Südwest durchgeführten Studie „Jugend, Information, Medien“ (JIM) weisen für die Altersgruppe der 12- bis 19-Jährigen auf eine hohe Verfügbarkeit von digitalen Endgeräten hin, die für Smartphones und Computer/Laptops im Jahr 2019 annährend 100 % betrug [2]. Dass digitale Medien nicht nur für freizeitbezogene Aktivitäten (Streaming, Musikhören, Spielen), sondern auch für gesundheitsbezogene Informationsanliegen genutzt werden, zeigen verschiedene Untersuchungen und Reviews [3–5].

In ihrer systematischen Übersichtsarbeit zur gesundheitsbezogenen Internetnutzung konnten Park und Kwon [3] 19 Studien mit insgesamt fast 11.000 Teilnehmenden (Alter bis 24 Jahre) identifizieren. Die Ergebnisse weisen darauf hin, dass ein Großteil das Internet schon einmal für gesundheitsbezogene Zwecke genutzt hat, wobei der PC/Laptop (65 %) sowie das Smartphone und andere mobile Endgeräte (42 %) häufig zur gesundheitsbezogenen Informationsrecherche eingesetzt wurden. Besonders bedeutsam waren hierbei alltagsnahe Gesundheitsthemen (z. B. Sportverletzungen, Fitness, Erkältung), körperliches Wohlbefinden, sexuelle Gesundheit, psychische Gesundheit oder soziale Probleme. Unterschiede in der gesundheitsbezogenen Internetnutzung ließen sich für das Alter (häufigere Nutzung mit zunehmendem Alter) und Geschlecht (häufigere Nutzung bei Mädchen, vor allem mit Blick auf das Hilfesuchverhalten) identifizieren. In einer Zusammenfassung der aktuellen Studienlage finden sich Hinweise für eine häufigere Nutzung klassischer Internetseiten und Suchmaschinen, während gesundheitsbezogene Apps und sogenannte Wearables deutlich seltener eingesetzt werden [4]. Eine zunehmende Bedeutung lässt sich hingegen für Social-Media-Plattformen (z. B. Youtube, Instagram) feststellen, die – wie eine Studie aus dem Vereinigten Königreich zeigt – häufig für Informationen zum Thema Körperbild, körperliche Aktivität und Fitness sowie Ernährung genutzt werden [5].

Wenn digitale Medien zunehmend für die gesundheitsbezogene Informationsrecherche zum Einsatz kommen, rückt die Frage nach der digitalen Gesundheitskompetenz (GK) im Jugendalter in den Vordergrund. Unter dem Begriff Gesundheitskompetenz wird grundlegend die Fähigkeit des Findens, Verstehens, der kritischen Bewertung und Anwendung gesundheitsbezogener Informationen zur Bewahrung, Förderung oder Wiederherstellung der Gesundheit und des Wohlbefindens verstanden [6]. Für den Bereich der digitalen GK (im Englischen auch als E‑Health Literacy oder Digital Health Literacy bezeichnet) liegt bislang keine einheitliche Definition vor, was unter anderem darauf zurückzuführen ist, dass unterschiedliche Konzepte wie das der GK mit weiteren Ansätzen wie der Medien- und Digitalkompetenz verbunden werden. Während „Digitalkompetenz“ als die Fähigkeit der angemessenen Nutzung von Medien- und Kommunikationstechnologien beschrieben wird, kann unter „digitaler Gesundheitskompetenz“ die Fähigkeit der angemessenen Nutzung von digitalen Informationstechnologien zur Erschließung und Verarbeitung gesundheitsbezogener Informationen verstanden werden [7]. Als diskursbestimmend galt dabei lange Zeit ein von Norman und Skinner vorgelegtes Modell, das 6 Fähigkeiten definiert, die sich einem analytischen Kompetenzbereich (Traditional Literacy, Information Media Literacy) und einem kontextspezifischen Fähigkeitsbereich (Computer Literacy, Science Literacy, Health Literacy) zuordnen lassen [8].

Trotz der Popularität, die das Modell sowie das von den Autoren entwickelte eHEALS-Instrument [9] erfahren haben, sind in den vergangenen Jahren u. a. die individuelle Fokussierung und die fehlende Berücksichtigung der sozialen Einbettung von Techniknutzung kritisiert worden [10]. Gerade mit Blick auf die zunehmende Bedeutung sozialer Medien als „user-generated platforms“ (d. h. Plattformen, in denen Nutzerinnen und Nutzer eigene Inhalte generieren können) sind (junge) Menschen nicht nur passive Rezipienten von gesundheitsbezogenen Informationen, sondern tragen durch eigene Inhalte oder durch Interaktion mit bestehenden Inhalten (Likes, Teilen und Kommentieren von Beiträgen) aktiv zum Informations- und Kommunikationsgeschehen bei.

Mit Blick auf aktuelle Befunde repräsentativer Studien variiert der Anteil der Erwachsenen (18+) aus Deutschland mit eingeschränkter digitaler GK von 52–75,8 % [11, 12]. Dabei berichten Befragte mit niedrigem Bildungs- sowie Sozialstatus, höherem Alter (65+) und geringer funktionaler Literalität deutlich häufiger von Schwierigkeiten der Beschaffung und des Umgangs mit digitalen Gesundheitsinformationen. Für das Kindes- und Jugendalter ist die empirische Datenlage bislang deutlich eingeschränkter. Während für die allgemeine GK mittlerweile verschiedene alters- und entwicklungsspezifische Instrumente und erste Studienbefunde vorliegen [13–16], existieren für den Bereich der digitalen GK national und international bisher kaum Studien. Im Rahmen der deutschen Übersetzung und psychometrischen Testung des eHEALS-Instruments wurden Jugendliche der gymnasialen Oberstufe (*n* = 326) zur digitalen GK befragt [17]. Dabei berichteten die Befragten am häufigsten von Schwierigkeiten, gesundheitsbezogene Entscheidungen auf Basis der im Internet gefundenen Informationen zu treffen (31,6 %) oder zu wissen, welche Quellen für gesundheitsbezogene Informationen im Internet verfügbar sind (20,2 %). In einer etwas älteren US-amerikanischen Untersuchung mit Jugendlichen der Klassenstufen 9 bis 12 ließ sich eine tendenziell hohe digitale GK (eHEALS-Score: 30,6 von 40) feststellen [18]. Unterschiede fanden sich hierbei zugunsten von älteren Jugendlichen und Befragten mit Erfahrung in der online- und offlinebezogenen Recherche nach Gesundheitsinformationen.

In Hinblick auf gesundheitliche Indikatoren ließen sich in einer türkischen Studie mit 14- bis 19-Jährigen unter Kontrolle von Geschlecht und Bildungsstand der Eltern Belege für positive Zusammenhänge zwischen digitaler GK (ebenfalls erfasst über den eHEALS) und dem Ernährungsverhalten, der sportlichen Aktivität und dem Stressmanagement ermitteln [19]. Levin-Zamir et al. berichten zudem die Ergebnisse einer israelischen Studie mit Schülerinnen und Schülern der Klassenstufen 7, 9 und 11, bei der ein eigenentwickeltes Instrument zu Media Health Literacy mit 4 Dimensionen zum Einsatz kam (Erkennen von Gesundheitsbotschaften in digitalen Medien; Einfluss von Medienbotschaften auf das eigene Verhalten; kritische Bewertung der Medienbotschaften; persönliche Handlungen als Folge der Medienbotschaften; [20]). Mädchen und Jugendliche aus Familien mit höherem mütterlichen Bildungsstand wiesen eine höhere digitale GK auf. Adjustiert für beide Variablen ließen sich positive Zusammenhänge zwischen der digitalen GK und dem gesundheitsförderlichen Verhalten absichern. Eine weitere Studie mit 12- bis 16-jährigen Jugendlichen aus China konnte zudem zeigen, dass die Fähigkeit der onlinebezogenen Beschaffung von Gesundheitsinformationen mit einer höheren Intention gesundheitsförderlichen Verhaltens assoziiert ist [21].

Für den deutschsprachigen Raum mangelt es bislang an Erkenntnissen zum Zusammenhang von digitaler GK und dem Gesundheitsverhalten. In einer vom Robert Koch-Institut durchgeführten repräsentativen Studie zur allgemeinen Gesundheitskompetenz von Jugendlichen zwischen 14 bis 17 Jahren wiesen Befragte mit geringen Ausprägungen in allen GK-Dimensionen eine höhere Wahrscheinlichkeit des nichttäglichen Obst- und Gemüsekonsums auf [14]. Auch ließen sich Zusammenhänge zwischen einer eingeschränkten Fähigkeit der Kommunikation und Interaktion sowie dem Rauchverhalten und einer geringen körperlichen Aktivität absichern.

Vor dem Hintergrund der begrenzten Studienlage zielt der vorliegende Beitrag darauf ab, erste Befunde zur digitalen GK bei Jugendlichen in Deutschland vorzustellen. Entsprechend der internationalen Studienlage gehen wir von geschlechts-, alters- und sozioökonomischen Unterschieden der digitalen Gesundheitskompetenz aus. Auch wird angenommen, dass sich die für die allgemeine GK [14] und digitale GK [19–21] identifizierten Zusammenhänge mit dem Gesundheitsverhalten auch für Jugendliche aus Deutschland nachweisen lassen. Aufgrund der unverändert hohen Prävalenz von Übergewicht und Adipositas im Jugendalter [22] stehen im Folgenden die körperliche Aktivität und Aspekte des Ernährungsverhaltens als Indikatoren des Gesundheitsverhaltens im Vordergrund.

## Methodik

### Studiendesign und Stichprobe

Um empirische Aussagen zur digitalen GK bei Jugendlichen treffen und Zusammenhänge mit sozialen Merkmalen und dem Gesundheitsverhalten prüfen zu können, wurde eine vom Hessischen Ministerium für Wissenschaft und Kunst (HMWK) geförderte Mixed-Methods-Studie an hessischen allgemeinbildenden Schulen durchgeführt. Diese bestand einerseits aus einer schriftlichen Befragung von Schülerinnen und Schülern der Sekundarstufe I sowie von Schulleitungen zu den Rahmenbedingungen an den jeweiligen Schulen (quantitativer Arm). Darüber hinaus wurden leitfadengestützte Einzelinterviews mit Lehrkräften in den für die schriftliche Befragung rekrutierten Schulen zu den Erfahrungen mit dem Thema GK im Kontext der Medienbildung durchgeführt (qualitativer Arm). Gegenstand des vorliegenden Beitrags ist der quantitative Teil der Studie, der auf einer Paper-Pencil-Befragung von Schülerinnen und Schülern im Zeitraum von Oktober 2019 bis Februar 2020 basiert.

Innerhalb der Landkreise Fulda und Wiesbaden wurden mit Ausnahme von Förderschulen alle vorhandenen Regelschulformen der Sekundarstufe I (Klassenstufe 8 und 9) zur Studienteilnahme eingeladen (*N* = 49). Die Einladung erfolgte zunächst per E‑Mail mit einem Informationsschreiben, welches über die Ziele, Themen und die geplante Umsetzung der Studie Auskunft gab. Dem folgten nach 2 Wochen ein Erinnerungsschreiben per E‑Mail und eine telefonische Nachfassung. Da nach Abschluss der Rekrutierung die Anzahl positiver Zusagen gering war (*n* = 10), wurden die Einzugsgebiete auf die angrenzenden Landkreise ausgeweitet (*N* = 100). Hierdurch konnten 6 weitere Schulen gewonnen werden, womit die Rücklaufquote insgesamt 10,7 % beträgt. Mit Einverständnis zur Studienteilnahme wurden den teilnehmenden Schulen die notwendigen Unterlagen (Fragebögen, Informationsschreiben, Einverständniserklärungen, Instruktionen) postalisch zugesandt. Die Befragung fand im Klassenkontext statt und wurde von einer zuvor instruierten Lehrkraft innerhalb einer Schulstunde durchgeführt. Voraussetzung der Befragungsteilnahme war das Vorliegen einer schriftlichen Einverständniserklärung der Schülerinnen und Schüler sowie der Erziehungsberechtigten bei Minderjährigkeit.

### Instrument

Zur Erfassung der *digitalen Gesundheitskompetenz* wurde das Digital Health Literacy Instrument (DHLI) von Van der Vaart und Drossaert [23] verwendet, zu dem bereits erste deutsche Anwendungserfahrungen vorliegen [11, 24]. Hierbei handelt es sich um ein Selbstbeurteilungsverfahren, welches verschiedene Facetten der digitalen GK erfasst. Von den im Rahmen der Originalversion vorgeschlagenen 7 Subskalen wurden innerhalb der vorliegenden Studie 5 Dimensionen eingesetzt: (1) Suchen und Finden von Informationen (z. B. richtige Suchanfragen verwenden), (2) Beitragen eigener Inhalte (z. B. eigene gesundheitliche Anliegen klar formulieren), (3) Bewertung der Qualität (z. B. mögliches kommerzielles Interesse von Gesundheitsinformationen erkennen), (4) Bestimmung der Alltagsrelevanz (z. B. Anwendung der Information im Alltag) und (5) Schutz der Privatsphäre (z. B. Teilen privater Informationen). Ausgeschlossen wurden hingegen die Subskalen „operative Fähigkeiten“ und „navigationale Fähigkeiten“, da diese aufgrund der eher technischen Ausrichtung (z. B. Fähigkeit zur Verwendung einer Tastatur) für Jugendliche als weniger relevant eingestuft wurden. Jede Subskala umfasst 3 Items, die auf einer vierstufigen Skala (1 = sehr schwierig, 2 = schwierig, 3 = einfach, 4 = sehr einfach bzw. für die Skala zum Schutz der Privatsphäre 1 = häufig, 2 = manchmal, 3 = selten, 4 = nie) beantwortet werden konnten (Items siehe Tab. 2).

Das englischsprachige Instrument wurde in einem Übersetzungs-Rückübersetzungs-Verfahren in die deutsche Sprache überführt, wobei stellenweise minimale Anpassungen vorgenommen wurden, um die Verständlichkeit für Jugendliche zu verbessern. Vor dem Einsatz wurde das Instrument in einem Pretest mit Jugendlichen auf Verständlichkeit hin erprobt. Mit einer Ausnahme erreichte die interne Konsistenz der eingesetzten Skalen zufriedenstellende Werte (0,65 < α < 0,75). Aufgrund der geringen Reliabilität (α = 0,29) wurde die Skala „Schutz der Privatsphäre“ aus den bi- und multivariaten Analysen ausgeschlossen.

Bisher existieren keine Grenzwerte zur Definition unterschiedlicher Kompetenzlevel, weshalb vorliegend eine inhaltlich begründete Kategorisierung vorgenommen wurde. In einem ersten Schritt wurde für jede Dimension ein Summenwert aller Items berechnet, woraus sich eine Spannweite von 3 (geringe digitale GK) bis 12 (hohe digitale GK) ergibt. Die anschließend vorgenommene Kategorisierung erfolgte unter der Annahme, dass Summenwerte von 3 bis 6 indizieren, dass die Befragten die jeweiligen Items als „schwierig“ oder „sehr schwierig“ bewerten (d. h. „niedrige digitale GK“). Hingegen wurde bei Werten von 9 bis 12 von einer Bewertung „sehr einfach“ und „einfach“ ausgegangen (d. h. hohe digitale GK). Summenwerte von 7 und 8 wurden entsprechend als „mittlere digitale GK“ kategorisiert.

Als Indikatoren des Gesundheitsverhaltens wurden das Ernährungsverhalten sowie die körperliche Aktivität in Anlehnung an die Health Behavior in School-aged Children (HBSC)-Studie erhoben. Das *Ernährungsverhalten* wurde über die wöchentliche Verzehrhäufigkeit von Obst, Gemüse und Softdrinks auf einer siebenstufigen Skala (1 = nie bis 7 = jeden Tag mehrmals) erfasst [25]. In Anlehnung an bestehende Ernährungsempfehlungen wurden die Items dichotomisiert, sodass der Anteil derjenigen Befragten mit nichttäglichem Konsum von Obst und Gemüse bzw. mit täglichem Konsum von Softdrinks ermittelt werden konnte. Zur Erfassung der *körperlichen Aktivität* wurden die Jugendlichen um eine Einschätzung gebeten, an wie vielen der letzten 7 Tage sie sich für mindestens 60 min körperlich angestrengt haben. Für die Auswertung wurde der Anteil der Befragten kategorisiert, die an weniger als 3 Tagen für mindestens 60 min körperlich aktiv waren (Kategorie „geringe körperliche Aktivität“; [25]). Insgesamt folgte die Kategorienbildung der Indikatoren des Ernährungs- und Bewegungsverhaltens mit dem Ziel der Vergleichbarkeit den HBSC-Berichterstattungsstandards [26].

Als soziodemografische Variablen wurden neben dem Geschlecht (männlich, weiblich, anderes Geschlecht) die Klassenstufe und die Schulform (bereits vorerfasst über die Codierung des Fragebogens) berücksichtigt. Zudem erfolgte die Messung des subjektiven Sozialstatus (SSS) mithilfe der deutschsprachigen Version der MacArthur-Scale [27]. Mittels einer 10-stufigen Skala (visualisiert in Form einer Leiter) wurden die Befragten gebeten, ihre Stellung innerhalb der Gesellschaft im Vergleich zu anderen Familien anzugeben. Für weitergehende Analysen wurden aus den in vorhergehenden Studien angewendeten Grenzwerten 3 Gruppen gebildet: niedriger SSS (1 bis 4), mittlerer SSS (5 bis 7) und hoher SSS (8 bis 10; [24]).

### Statistische Analysen

Zur Erfassung systematischer Ausfälle auf Itemebene wurde zunächst der Anteil fehlender Werte für alle Items der digitalen GK ermittelt. Mit einem prozentualen Anteil von 2,2–4,3 % ließen sich keine besonderen Muster feststellen. Die univariate Auswertung der Daten zur digitalen GK und zum Gesundheitsverhalten erfolgte unter Zuhilfenahme von absoluten und relativen Häufigkeiten. Um Unterschiede der digitalen GK in Abhängigkeit von soziodemografischen Merkmalen zu untersuchen, wurden in einem zweiten Schritt Kreuztabellen mit angeschlossenem Chi-Quadrat-Test (χ^2^) berechnet. In Fällen mit Zellbesetzungen unter *n* = 5 wurde auf den exakten Test nach Fisher (FET) zurückgegriffen. Für die χ^2^-Unabhängigkeitstests und den exakten Test nach Fisher wurde ein Signifikanzniveau von *p* < 0,050 festgelegt. In einem letzten analytischen Schritt wurden binärlogistische Regressionsmodelle berechnet, um Assoziationen zwischen den Dimensionen der digitalen GK und den Indikatoren des Gesundheitsverhaltens zu ermitteln (getrennte Modelle für die einzelnen Indikatoren des Gesundheitsverhaltens). Als Referenzkategorie diente eine hohe Ausprägung der digitalen GK in den jeweiligen Dimensionen. Dabei wurde für das Geschlecht und den SSS adjustiert, da sich diese Variablen in den bivariaten Analysen für die Ausprägung der digitalen GK als statistisch bedeutsam erwiesen. Ausgewiesen werden die Odds Ratios (OR) als Chancenverhältnisse sowie die dazugehörigen Konfidenzintervalle (95 %-KI). Signifikante Assoziationen wurden entlang der Signifikanzniveaus (*p* < 0,050 bis *p* < 0,001) berichtet. Alle Berechnungen wurden mit der Statistiksoftware IBM SPSS Statistics 25 (IBM Corp., Armonk, NY, USA) vorgenommen.

## Ergebnisse

### Beschreibung der Stichprobe und Verteilung zentraler Untersuchungsmerkmale

Von den 16 rekrutierten Schulen konnte die Befragung in 3 Schulen aufgrund zeitlicher Restriktionen nicht umgesetzt werden. Auch eine Nacherhebung war aufgrund der beginnenden COVID-19-Pandemie nicht möglich. Nach Plausibilitätskontrolle und Bereinigung der Daten umfasst die finale Stichprobe 490 Schülerinnen und Schüler aus 13 Schulen. Mit einem Anteil männlicher Befragten von 51,2 % und weiblicher Befragten von 47,1 % erweist sich die Geschlechterverteilung als ausgeglichen (Tab. 1). Ein ähnliches Bild zeigt sich hinsichtlich der Verteilung über die Klassenstufen 8 (48,0 %) und 9 (52,0 %). 41,8 % der Befragten besuchen das Gymnasium, während ein weiteres Drittel (35,1 %) die Gesamtschule und ein Viertel (23,1 %) die Realschule besucht. Ihren SSS bewerten 4,8 % der Schülerinnen und Schüler als gering und 30,9 % als hoch. Bezüglich der Indikatoren des Gesundheitsverhaltens ist ein Drittel in einem geringen Ausmaß körperlich aktiv. Während die tägliche Verzehrhäufigkeit von Obst und Gemüse bei 42,4 % bzw. 34,2 % liegt, geben 12,6 % der Befragten an, täglich zuckerhaltige Getränke wie Cola zu konsumieren. Die vollständige Beschreibung der Stichprobe und der zentralen Untersuchungsmerkmale findet sich in Tab. 1.

**Table 1:** 

	*n*	%
*Geschlecht*		
Männlich	251	51,2
Weiblich	231	47,1
Anderes Geschlecht	8	1,6
*Klassenstufe*		
Klasse 8	235	48,0
Klasse 9	255	52,0
*Schulform*		
Realschule	113	23,1
Gesamtschule	172	35,1
Gymnasium	205	41,8
*Subjektiver Sozialstatus*		
Hoch	148	30,9
Mittel	308	64,3
Niedrig	23	4,8
*Körperliche Aktivität*		
< 3 Tage für jeweils mind. 60 min	159	32,7
≥ 3 Tage für jeweils mind. 60 min	327	67,3
*Verzehr von Obst*		
Täglich	206	42,4
Nicht täglich	280	57,6
*Verzehr von Gemüse*		
Täglich	166	34,2
Nicht täglich	319	65,8
*Verzehr von zuckerhaltigen Getränken*		
Täglich	61	12,6
Nicht täglich	423	87,4
**Gesamt**	**490**	**100**

### Ausprägung der digitalen Gesundheitskompetenz

In Tab. 2 sind die prozentualen Häufigkeiten der jeweils geringen Ausprägungen der Einzelitems des DHLI dargestellt (Antwortangaben „schwierig/sehr schwierig“ bzw. „manchmal/häufig“). Dabei reicht die Spannweite der Jugendlichen, die über Schwierigkeiten berichten, von 15,3–37,5 %. Innerhalb der Dimension „Suchen und Finden von Informationen“ berichten die Jugendlichen mit 31,4 % die größten Schwierigkeiten im Finden der richtigen Informationen, während die Verwendung passender Begriffe oder Suchanfragen von 17,4 % als (sehr) schwierig bewertet wird. Für die Dimension „Beitragen eigener Inhalte“ erweist sich das schriftliche Ausdrücken der eigenen Meinung, Gefühle und Gedanken für annähernd ein Viertel der Befragten (24,5 %) als (sehr) schwierig, während innerhalb der Dimension „Bewertung der Qualität“ das Erkennen kommerzieller Interessen oder einer qualitativ hochwertigen Gesundheitsinformation von jeweils 32,3 % als (sehr) schwierig bewertet wird. Auch berichten jeweils 30,2 % der Jugendlichen über Schwierigkeiten, anhand der Informationen eine Entscheidung für die eigene Gesundheit treffen zu können oder diese im Alltag anzuwenden (Dimension „Bestimmung der Alltagsrelevanz“). Schließlich findet sich für die Dimension „Schutz der Privatsphäre“ die am häufigsten berichtete Schwierigkeit in der Beurteilung, wer die Informationseingaben der Jugendlichen mitlesen kann (37,5 %).

**Table 2:** 

Nr.	Frage	% (95 %-KI)
*Informationssuche*: Wenn du im Internet nach Informationen zum Thema Gesundheit suchst, wie einfach oder schwierig ist es für dich, …		
1	… genau die richtigen Informationen zu finden, die du suchst?	31,4
		(27,2–35,7)
2	… eine Auswahl aus allen Informationen zu treffen, die du findest?	24,9
		(21,1–28,8)
3	… die passenden Begriffe oder Suchanfragen zu verwenden, um die gesuchten Informationen zu finden?	17,4
		(14,0–20,8)
*Beitragen eigener Inhalte*: Wenn du eine Nachricht schreibst (z. B. in einem Forum oder in sozialen Netzwerken wie Facebook oder Twitter), wie einfach oder schwierig ist es für dich, …		
4	… deine Meinungen, Gedanken und Gefühle schriftlich auszudrücken?	24,5
		(20,7–28,4)
5	… deine Nachricht so zu schreiben, dass andere Menschen genau verstehen, was du meinst?	21,5
		(18,0–25,0)
6	… deine Frage oder gesundheitliches Anliegen klar zu formulieren?	17,1
		(13,3–20,7)
*Zuverlässigkeit bewerten*: Wenn du im Internet nach Informationen zum Thema Gesundheit suchst, wie einfach oder schwierig ist es für dich, …		
7	… zu entscheiden, ob eine Information von hoher Qualität ist?	32,3
		(28,2–36,5)
8	… zu entscheiden, ob eine Information mit finanziellen Interessen geschrieben worden ist (z. B. von Personen, die ein Produkt verkaufen möchten)?	32,1
		(28,1–36,7)
9	… verschiedene Internetseiten zu überprüfen, um zu sehen, ob sie die gleichen Informationen bereitstellen?	15,3
		(12,1–18,7)
*Bestimmung der Relevanz*: Wenn du im Internet nach Informationen zum Thema Gesundheit suchst, wie einfach oder schwierig ist es für dich, …		
10	… die Informationen, die du gefunden hast, in deinem Alltag anzuwenden?	30,2
		(26,0–34,3)
11	… die Informationen zu nutzen, die du gefunden hast, um Entscheidungen über deine Gesundheit zu treffen (z. B. Ernährung, Sport)?	29,1
		(24,8–33,5)
12	… zu entscheiden, ob die Informationen, die du gefunden hast, auf dich zutreffen?	19,1
		(15,7–22,8)
*Schutz der Privatsphäre*: Wenn du eine Nachricht in einem Forum oder in den sozialen Netzwerken postest, wie oft …		
13	… findest du es schwierig zu beurteilen, wer mitlesen kann, was du schreibst?	37,5
		(33,3–41,8)
14	… teilst du (absichtlich oder unabsichtlich) private Informationen über dich (z. B. Name oder Adresse)?	19,9
		(16,4–23,6)
15	… teilst du (absichtlich oder unabsichtlich) private Informationen einer anderen Person?	16,7
		(13,5–20,2)

Dargestellt sind die prozentualen Häufigkeiten der Antwortoptionen „schwierig/sehr schwierig“ bzw. (für die Skala „Schutz der Privatsphäre“) „manchmal/häufig“

*KI* Konfidenzintervall

Stratifiziert nach sozialen Merkmalen finden sich für das Geschlecht und den SSS Unterschiede in der Ausprägung der digitalen GK (Tab. 3). Dabei weisen Schüler im Vergleich zu ihren Mitschülerinnen häufiger eine stärker ausgeprägte Fähigkeit des Suchens und Findens von Informationen (χ^2^(1) = 9,07, *p* < 0,050), des Beitragens eigener Inhalte (χ^2^(1) = 6,73, *p* < 0,050) und der Bewertung der Qualität von digitalen Gesundheitsinformationen auf (χ^2^(1) = 9,37, *p* < 0,010). Während sich differenziert nach Klassenstufe und Schulform keine Unterschiede statistisch absichern lassen, erweist sich der SSS für die Ausprägung der digitalen GK als relevant. Über alle Subdimensionen hinweg berichten Jugendliche mit einem geringen SSS häufiger von Schwierigkeiten im Suchen und Finden (FET = 9,94, *p* < 0,050), im Beitragen eigener Inhalte (FET = 12,81, *p* < 0,010), in der Bewertung der Qualität (FET = 15,88, *p* < 0,001) und der Bestimmung der Alltagsrelevanz (χ^2^(2) = 19,14, *p* < 0,010) von digitalen Gesundheitsinformationen.

**Table 3:** 

	Informationssuche			Beitragen eigener Inhalte			Zuverlässigkeit bewerten			Bestimmung der Relevanz		
	Hoch % (*n*)	Mittel % (*n*)	Gering % (*n*)	Hoch % (*n*)	Mittel % (*n*)	Gering % (*n*)	Hoch % (*n*)	Mittel % (*n*)	Gering % (*n*)	Hoch % (*n*)	Mittel % (*n*)	Gering % (*n*)
*Geschlecht*	χ2 = 9,07 (1)*, p* < 0,05			χ2 = 6,73 (1)*, p* < 0,05			χ2 = 9,37 (1)*, p* < 0,01			n. s.		
Männlich	63,3 % (155)	31,4 % (77)	5,3 % (13)	68,9 % (162)	26,4 % (62)	4,7 % (11)	62,8 % (150)	30,5 % (73)	6,7 % (16)	57,2 % (135)	33,9 % (80)	8,9 % (21)
Weiblich	50,2 % (113)	40,0 % (90)	9,8 % (22)	61,9 % (135)	27,1 % (59)	11,0 % (24)	48,9 % (109)	43,5 % (97)	7,6 % (17)	50,5 % (109)	41,2 % (89)	8,3 % (18)
*Klassenstufe*	n. s.			n. s.			n. s.			n. s.		
Klasse 8	59,7 % (136)	35,5 % (81)	4,8 % (11)	69,2 % (150)	25,3 % (55)	5,5 % (12)	54,7 % (123)	37,7 % (85)	7,6 % (17)	53,4 % (118)	38,9 % (86)	7,7 % (17)
Klasse 9	56,0 % (140)	34,4 % (86)	9,6 % (24)	63,1 % (154)	27,5 % (67)	9,4 % (23)	58,4 % (143)	35,1 % (86)	6,5 % (16)	55,7 % (133)	34,7 % (83)	9,6 % (23)
*Schulform*	n. s.			n. s.			n. s.			n. s.		
Realschule	58,2 % (64)	31,8 % (35)	10,0 % (11)	59,8 % (64)	28,1 % (30)	12,1 % (13)	55,5 % (60)	38,0 % (41)	6,5 % (7)	51,8 % (56)	38,0 % (41)	10,2 % (11)
Gesamtschule	51,8 % (86)	41,6 % (69)	6,6 % (11)	68,1 % (111)	25,8 % (42)	6,1 % (10)	52,5 % (85)	38,9 % (63)	8,6 % (14)	59,8 % (95)	32,7 % (52)	7,5 % (12)
Gymnasium	62,4 % (126)	31,2 % (63)	6,4 % (13)	67,5 % (129)	26,2 % (50)	6,3 % (12)	60,5 % (121)	33,5 % (67)	6,0 % (12)	51,8 % (100)	39,4 % (76)	8,8 % (17)
*Subjektiver Sozialstatus*	*FET* *=* *9,94, p* < 0,05			FET = 12,81*, p* < 0,01			FET = 15,88*, p* < 0,01			χ2 = 19,14 (2)*, p* < 0,001		
Hoch	66,6 % (96)	29,2 % (42)	4,2 % (6)	75,4 % (104)	17,4 % (24)	72 % (10)	68,8 % (95)	26,9 % (37)	4,3 % (6)	65,2 % (90)	29,0 % (40)	5,8 % (8)
Mittel	54,8 % (165)	36,6 % (110)	8,6 % (26)	63,5 % (186)	29,0 % (85)	7,5 % (22)	51,8 % (156)	40,9 % (123)	7,3 % (22)	50,9 % (149)	40,6 % (119)	8,5 % (25)
Niedrig	40,9 % (9)	45,5 % (10)	13,6 % (3)	42,9 % (9)	42,9 % (9)	14,2 % (3)	57,2 % (12)	23,8 % (5)	19,0 % (4)	38,1 % (8)	33,3 % (7)	28,6 % (6)
**Gesamt**	57,8 % (276)	34,9 % (176)	7,3 % (35)	65,9 % (304)	26,5 % (122)	7,6 % (35)	56,6 % (266)	36,4 % (171)	7,0 % (33)	54,6 % (251)	36,7 % (169)	8,7 % (40)

Einteilung in hohe, mittlere und geringe digitale GK anhand des Summenscores (hohe dGK: 9–12, mittlere dGK: 7–8, geringe dGK: 3–6), Dimension „Schutz der Privatsphäre“ aufgrund von geringer interner Konsistenz aus der Analyse ausgeschlossen

*n* Häufigkeit, *n.* *s.* nicht signifikant, *χ*^*2*^ Chi-Quadrat, *FET* exakter Test nach Fisher

### Zusammenhänge der digitalen Gesundheitskompetenz mit dem Gesundheitsverhalten

Die in Tab. 4 dargestellten Ergebnisse der binärlogistischen Regressionen zeigen zusammenfassend, dass das Gesundheitsverhalten in Abhängigkeit von der Ausprägung einzelner Dimensionen der digitalen GK variiert. Für eine geringe körperliche Aktivität erweisen sich eine mittlere und eine geringe Fähigkeit der Bestimmung der Alltagsrelevanz digitaler Gesundheitsinformationen als statistisch bedeutsam (mittlere digitale GK: OR = 1,74, *p* < 0,050; geringe digitale GK: OR = 2,37, *p* < 0,050). Hingegen geht eine mittlere Fähigkeit des Suchens und Findens von digitalen Gesundheitsinformationen auch nach Kontrolle von Geschlecht und SSS mit einer erhöhten Wahrscheinlichkeit eines nichttäglichen Obstkonsums (OR = 1,63, *p* < 0,050) einher. Während der nichttägliche Verzehr von Gemüse mit keiner Dimension der digitalen GK assoziiert ist, finden sich für den täglichen Konsum von zuckerhaltigen Getränken signifikante Zusammenhänge mit einer mittleren Fähigkeit des Beitragens eigener Inhalte (OR = 2,45, *p* < 0,010).

**Table 4:** 

	Geringe körperliche Aktivität		Nichttäglicher Verzehr von Obst		Nichttäglicher Verzehr von Gemüse		Täglicher Verzehr von zuckerhaltigen Getränken	
	%	OR (95 %-KI)	%	OR (95 %-KI)	%	OR (95 %-KI)	%	OR (95 %-KI)
*Suchen und Finden*								
Hoch (Ref.)	31,1	1,00	51,6	1,00	65,2	1,00	10,9	1,00
Mittel	34,3	1,02 (0,62–1,69)	65,7	1,63 (1,01–2,63)*	65,7	0,91 (0,56–1,50)	14,0	0,84 (0,42–1,69)
Gering	40,0	1,48 (0,61–3,61)	71,4	1,99 (0,80–5,01)	67,6	0,69 (0,28–1,71)	23,5	1,70 (0,60–4,86)
*Beitragen eigener Inhalte*								
Hoch (Ref.)	33,6	1,00	54,6	1,00	62,3	1,00	8,6	1,00
Mittel	28,7	0,61 (0,36–1,03)	59,8	0,92 (0,56–1,50)	70,2	1,44 (0,86–2,41)	23,3	2,45 (1,29–4,65)**
Gering	34,3	0,63 (0,28–1,44)	68,6	1,47 (0,65–3,34)	74,3	2,07 (0,87–4,91)	17,1	1,42 (0,47–4,33)
*Bewertung der Qualität*								
Hoch (Ref.)	32,6	1,00	54,3	1,00	64,2	1,00	9,1	1,00
Mittel	34,5	0,86 (0,52–1,43)	61,2	1,06 (0,65–1,72)	64,1	0,89 (0,54–1,47)	18,8	1,85 (0,93–3,70)
Gering	21,2	0,41 (0,15–1,12)	66,7	1,24 (0,50–3,09)	75,0	1,48 (0,55–3,98)	15,6	1,35 (0,39–4,64)
*Bestimmung der Relevanz*								
Hoch (Ref.)	27,6	1,00	52,0	1,00	61,6	1,00	11,2	1,00
Mittel	39,3	1,74 (1,05–2,86)*	62,7	1,23 (0,76–1,98)	72,0	1,45 (0,88–2,40)	15,5	1,08 (0,55–2,14)
Gering	45,0	2,37 (1,02–5,50)*	74,4	1,88 (0,76–4,62)	64,1	0,89 (0,38–2,08)	15,4	1,12 (0,36–3,42)

OR adjustiert nach Geschlecht und subjektiven Sozialstatus (SSS), Einteilung in hohe, mittlere und geringe digitale GK anhand des Summenscores (hohe dGK: 9–12, mittlere dGK: 7–8, geringe dGK: 3–6), Dimension „Schutz der Privatsphäre“ aufgrund von geringer interner Konsistenz aus der Analyse ausgeschlossen

*OR* Odds Ratio, *KI* Konfidenzintervall, *Ref.* Referenzgruppe

*p < 0,05, **p < 0,01

## Diskussion

Infolge der zunehmenden Verfügbarkeit und Nutzung digitaler Endgeräte hat auch die Bedeutung digitaler Gesundheitsinformationen spürbar zugenommen. Deren Relevanz dürfte im Zuge der COVID-19-Pandemie noch einmal deutlich gestiegen sein, wobei die Informationsmenge insbesondere in sozialen Medien auch mit einer Zunahme von gesundheitsbezogenen Miss- und Desinformationen einhergeht [28]. Aufgrund dieses auch als „Infodemie“ bezeichneten Phänomens bedarf es umso mehr der Fähigkeit, geeignete gesundheitsbezogene Informationen in den hochkomplexen und -dynamischen (digitalen) Informationswelten zu beschaffen und angemessen zu nutzen [29].

### Bewertung der Qualität von digitalen Gesundheitsinformationen

Die Ergebnisse der vorliegenden Studie liefern erste Erkenntnisse zur digitalen GK von Jugendlichen der Klassenstufen 8 und 9. Über alle Items hinweg berichten Jugendliche tendenziell die meisten Schwierigkeiten in der Bewertung der Qualität sowie der Bestimmung der Alltagsrelevanz von digitalen Gesundheitsinformationen. Diese Ergebnisse decken sich mit den Befunden einer vorangegangenen repräsentativen Studie mit Erwachsenen, bei der ebenfalls das DHLI zum Einsatz kam [12]. Auch erwies sich die Fähigkeit der Qualitätsbewertung in einer frühen Phase der COVID-19-Pandemie bei mehr als einem Drittel der in der COVID-HL-Studie befragten Studierenden als eingeschränkt [24]. Vor dem Hintergrund der zunehmenden digitalen Informationsvielfalt ist dieser Studienbefund wenig überraschend, aber umso relevanter.

Ergebnisse einer von der Vodafone Stiftung Deutschland im Jahr 2020 durchgeführten Studie zeigen, dass drei Viertel der 14- bis 24-Jährigen mindestens wöchentlich Kontakt mit Falschnachrichten haben, was gegenüber dem Jahr 2018 einer Zunahme von etwa 25 % entspricht [30]. Dabei gab etwa ein Drittel der Befragten an, dass es ihnen schwerfällt, glaubwürdige von unglaubwürdigen Informationen zu unterscheiden. Die vorliegenden Befunde zur Fähigkeit Jugendlicher, die Qualität von digitalen Gesundheitsinformationen zu bewerten, ordnen sich konsistent in diese Befundlage ein.

Weitere Einblicke in die Fähigkeit der Qualitätsbewertung von onlinebasierten Gesundheitsinformationen bei 13- bis 18-Jährigen geben Freeman et al. [31] in ihrer systematischen Übersichtsarbeit. Anhand der einbezogenen Studien ließen sich 4 übergeordnete Bewertungskategorien identifizieren: (1) Bewertung anhand der Bezeichnung der Website und deren Reputation (z. B. höhere Glaubwürdigkeit bei Domainendungen wie .org oder .edu), (2) Bewertung anhand der ersten Eindrücke beim Besuch der Website (z. B. gut strukturiert, prägnant, klar, gut verständlich), (3) Bewertung der Inhalte einer Website (z. B. durch Abgleich mit Inhalten anderer Webseiten) und (4) Fehlen einer systematischen Bewertung (z. B. nach Gefühl, unter Rückgriff der ersten Treffer in Suchmaschinen). Diese Erkenntnisse bieten sinnvolle Erklärungen, die im Rahmen von Maßnahmen zur Stärkung der Fähigkeit der Qualitätsbewertung einfließen können.

### Anwendung von digitalen Gesundheitsinformationen

Bei der Interpretation der Ergebnisse zur Bestimmung der Alltagsrelevanz ist zu berücksichtigen, dass die Anwendung onlinebezogener Gesundheitsinformationen eine nicht zu unterschätzende Herausforderung darstellt. So setzt der Handlungstransfer voraus, dass zuvor geeignete Informationen gefunden und verstanden sowie für die eigene Lebenssituation als relevant bewertet werden. Selbst wenn diese Voraussetzungen erfüllt sind, ist die Übersetzung von Wissen und Intentionen in tatsächliches Verhalten von zahlreichen weiteren (individuellen und umweltbezogenen) Faktoren abhängig [32–34].

Konsistent mit den Befunden zur allgemeinen GK [11, 12, 15] ließ sich in der vorliegenden Studie erstmals auch für die digitale GK über alle erfassten Dimensionen ein sozialer Gradient zuungunsten von Jugendlichen mit geringem SSS identifizieren. Aufgrund ihrer Bedeutung für das gesundheitliche Verhalten und weitere Gesundheitsoutcomes ist in weiteren Längsschnittstudien zu prüfen, ob die Förderung der digitalen GK einen Beitrag zur Reduzierung sozialbedingter Ungleichheiten von Gesundheit leisten kann.

Hingegen fällt die geschlechtsspezifische Befundlage in der Literatur deutlich heterogener aus. Während sich sowohl in den Ergebnissen der HBSC-Studie für 3 der 10 untersuchten Länder [15] sowie in einer israelischen Studie [20] Unterschiede in der (digitalen) GK zugunsten der Mädchen ergaben, ließen sich in 2 im Bundesland Nordrhein-Westfalen durchgeführten Surveys keine Geschlechtsunterschiede feststellen [35, 36]. Ob die in der vorliegenden Studie zuungunsten der Mädchen identifizierten Unterschiede generalisiert werden können, ist in größeren und repräsentativ angelegten Untersuchungen zu überprüfen.

Ebenfalls konnten entgegen der in vorherigen Studien [18, 20] festgestellten Unterschiede zugunsten älterer Jugendlicher vorliegend keine Unterschiede festgestellt werden. Dabei ist zu berücksichtigen, dass das Alter in dieser Studie über die Klassenstufe operationalisiert wurde. Zum einen ist das Alter innerhalb einer Klassenstufe heterogen und zum anderen ist die gesamte über die Klassenstufe 8 und 9 abgebildete Altersspanne möglicherweise zu gering, um Unterschiede erfassen zu können.

### Assoziationen von Gesundheitskompetenz mit dem Ernährungs- und Bewegungsverhalten

Schließlich ließen sich in dieser Untersuchung unter Kontrolle von Geschlecht und SSS signifikante Assoziationen zwischen den Dimensionen der digitalen GK und dem Ernährungs- und Bewegungsverhalten Jugendlicher feststellen. Damit bestätigen die Befunde die bereits für die allgemeine GK beobachteten Zusammenhänge mit Indikatoren des Gesundheitsverhaltens [14, 35, 37]. Dabei weisen die vorliegenden Befunde auf differenzielle Muster hin. Während die körperliche Aktivität signifikant mit der Fähigkeit der Relevanzbestimmung assoziiert war, erwiesen sich zur Vorhersage des Konsums von Obst und zuckerhaltigen Getränken die Fähigkeiten des Suchens und Findens sowie der Kommunikation (Beitragen eigener Inhalte) als bedeutsam. Hingegen ließen sich für den Gemüsekonsum keine signifikanten Zusammenhänge mit den untersuchten Dimensionen der digitalen GK absichern.

Diese Ergebnisse sind aus verschiedenen Gründen bedeutsam: Während in bisherigen Studien vor allem ein übergeordneter Globalfaktor der GK betrachtet wurde, legen die vorliegenden Ergebnisse die Notwendigkeit eines differenzierten Blicks auf einzelne Dimensionen von (digitaler) GK nahe. So sind das dimensionale Binnenverhältnis der GK und ihre Bedeutung für gesundheitliche Indikatoren mit einigen Ausnahmen [14, 38] bislang kaum Gegenstand der Forschung. Eine solche Differenzierung wäre aber für die Entwicklung möglichst spezifischer Interventionen nötig. Zum anderen weisen die Ergebnisse zumindest partiell auf die Relevanz der Fähigkeit des Beitragens eigener Inhalte und damit auf die notwendige Erweiterung bestehender GK-Konzepte um Interaktions- und Kommunikationskompetenzen hin [11, 14]. Ob die Stärkung der digitalen Gesundheitskompetenz im Jugendalter zu einer Förderung des Bewegungs- und Ernährungsverhaltens beitragen kann, ist zukünftig im Rahmen von Interventions- und Längsschnittstudien zu untersuchen. In einer systematischen Übersichtsarbeit von Interventionen zur Förderung der Gesundheitskompetenz im Erwachsenenalter ließ sich für die Mehrheit der Studien (7/8) eine Verbesserung von Indikatoren des Gesundheitsverhaltens im Prä-Post-Vergleich feststellen [39].

### Stärken und Limitation

Die vorliegende Studie liefert erste Befunde zur digitalen GK bei Jugendlichen in Deutschland und leistet somit einen Beitrag zur Schließung einer bislang bestehenden Forschungslücke. Dabei kam mit dem DHLI ein neuartiges Instrument zum Einsatz, welches die Dimension des Findens, Verstehens, der kritischen Bewertung und Anwendung um eine kommunikative und datenschutzbezogene Perspektive erweitert. Einschränkend ist anzumerken, dass im Rahmen der Übersetzung lediglich minimale sprachliche Anpassung vorgenommen und Jugendliche in diesen Prozess nicht systematisch einbezogen wurden. Jüngste Ergebnisse einer qualitativen Studie mit 34 Jugendlichen im Alter von 10 bis 18 Jahren erbrachten verschiedene sprachliche Anpassungsbedarfe [40]. Diese Befunde bieten sinnvolle Anknüpfungspunkte für die weitere Adaptierung des DHLI an die Zielgruppe der Jugendlichen.

Zudem ist zu berücksichtigen, dass zur Kategorisierung der digitalen GK nicht auf etablierte Grenzwerte zurückgegriffen werden konnte. Entgegen einer datengeleiteten Dichotomisierung, die u. a. mit dem Risiko des Informationsverlusts einhergeht [41], wurde vorliegend eine inhaltlich begründete Einteilung in 3 Gruppen (hohe, mittlere und geringe digitale GK) vorgenommen. Eine Überprüfung und Weiterentwicklung dieser Kategorisierung ist in künftigen Studien anzustreben. Darüber hinaus ist auf das Querschnittsdesign der Studie hinzuweisen, womit die Ergebnisse keine kausalen Rückschlüsse erlauben. Neben Längsschnittstudien wären in Zukunft repräsentative Untersuchungen zur digitalen GK bei Jugendlichen anzustreben. Wünschenswert wären dabei auch größere Stichproben, welche insbesondere bei multivariaten Berechnungen Ergebnisse besser absichern könnten und Kategorisierungen seltener erforderlich machen. Dabei sollte in künftigen Studien auch der hierarchischen Datenstruktur (Schüler*innen, Klasse, Schule) stärker Rechnung getragen werden, was die Erfassung der Gruppenzugehörigkeit und eine entsprechend große Fallzahl voraussetzt. Aufgrund der vergleichsweise geringen Stichprobengröße in der vorliegenden Studie kam es zum Teil zu geringen Gruppengrößen, was bei der Interpretation der Ergebnisse berücksichtigt werden muss.

Hiermit einhergehend ist auf den vergleichsweise geringen Rücklauf hinzuweisen, was das Potenzial von Verzerrungen durch systematische Ausfälle erhöht. Zwar wurden verschiedene Maßnahmen zur Erhöhung der Ausschöpfung unternommen (Variation in der Kontaktart, wiederholte Kontaktierung, kostenfreier Rückversand), jedoch ist zu berücksichtigen, dass der Aufwand für Schulen durch die Teilnahme an der schriftlichen Befragung und den Einzelinterviews vergleichsweise hoch ausfiel.

### Fazit und Implikationen

Zusammenfassend lässt sich anhand der Ergebnisse dieser Studie ein Handlungsbedarf insbesondere für die Dimensionen der Qualitätsbewertung und der Anwendung von digitalen Gesundheitsinformationen ableiten. Dabei sind Interventionen auf die Bedürfnisse von jungen Menschen mit geringem sozioökonomischen Status auszurichten und in den zentralen Lebenswelten von Jugendlichen zu verorten. Die Schule ist als Lehr- und Lernort ein zentrales Setting zur Förderung von (digitaler) GK, dies auch deshalb, da Medien- und Digitalkompetenzen eine zentrale Strategie der Kultusministerkonferenz darstellen, die auf Ebene der Bundesländer in Medienkompetenzrahmen überführt wurden. Dabei weisen beide Konzepte eine große Passung auf, d. h., digitale GK ließe sich leicht in bestehende Medienkompetenzrahmen integrieren [42]. Dabei ist im Sinne der relationalen Ausrichtung zu berücksichtigen, dass die individuelle digitale GK von den systemischen Strukturen und Rahmenbedingungen determiniert wird. Auf Ebene von Schule zu nennen sind die digitale Medieninfrastruktur sowie die Vermittlungskompetenzen von Lehrkräften. Dazu gehört auch, dass digitale Informationsanbieter (z. B. soziale Medien) Bedingungen schaffen, unter denen Jugendliche gesundheitsbezogene Informationen gut finden und hinsichtlich ihrer Qualität bewerten können (z. B. durch Markierung fehlerhafter oder irreführender Informationen).

## References

[1] Kirschner PA, De Bruyckere P (2017). The myths of the digital native and the multitasker. Teach Teach Educ.

[2] Feierabend S, Rathgeb T, Reutter T (2019) JIM-Studie 2019. Jugend, Information, Medien. Medienpädaogischer Forschungsverbund Südwest. https://bit.ly/3DqGjZN. Zugegriffen: 17. Nov. 2021

[3] Park E, Kwon M (2018). Health-related internet use by children and adolescents: systematic review. J Med Internet Res.

[4] Lupton D (2021). Young people’s use of digital health technologies in the global north: narrative review. J Med Internet Res.

[5] Goodyear VA, Armour KM, Wood A (2019). Young people and their engagement with health-related social media: new perspectives. Sport Educ Soc.

[6] Sørensen K, Van den Broucke S, Fullam J (2012). Health literacy and public health: a systematic reviewand integration of definitions and models. BMC Public Health.

[7] van Kessel R, Wong BLH, Clemens T, Brand H (2022). Digital health literacy as a super determinant of health: more than simply the sum of its parts. Internet Interv.

[8] Norman CD, Skinner HA (2006). ehealth literacy: essential skills for consumer health in a networked world. J Med Internet Res.

[9] Norman CD, Skinner HA (2006). eHEALS: the ehealth literacy scale. J Med Internet Res.

[10] Bittlingmayer U, Dadaczynski K, Sahrai D, van den Broucke S, Okan O (2020). Digitale Gesundheitskompetenz – Konzeptionelle Verortung, Erfassung und Förderung mit Fokus auf Kinder und Jugendliche. Bundesgesundheitsblatt Gesundheitsforschung Gesundheitsschutz.

[11] Kolpatzik K, Mohrmann M, Zeeb H (2020). Digitale Gesundheitskompetenz in Deutschland.

[12] Schaefer D, Gille S, Berens E-M, Griese L, Klinger J, Vogt D, Hurrelmann K (2021). Digitale Gesundheitskompetenz der Bevölkerung in Deutschland. Ergebnisse des HLS-GER 2. Gesundheitswesen.

[13] Bollweg TM, Okan O, Fretian A, Bröder J, Domanska O, Jordan S, Bruland D, Pinheiro P, Bauer U (2020). Adapting the European health literacy survey questionnaire for fourth-grade students in Germany: validation and psychometric analysis. Health Lit Res Pract.

[14] Domanska OM, Loer A-KM, Stock C, Jordan S (2021). Gesundheitskompetenz und Gesundheitsverhalten im Jugendalter: Ergebnisse einer bundesweiten Online-Befragung Jugendlicher. Präv Gesundheitsf.

[15] Paakkari L, Torppa M, Mazur J, Boberova Z, Sudeck G, Kalman M, Paakkari O (2020). A comparative study on adolescents’ health literacy in Europa: findings from the HBSC study. Int J Environ Res Public Health.

[16] Teufl L, Vrtis D, Felder-Puig R (2020). QUIGK-K: Quiz zur Erhebung von Gesundheitskompetenz bei Kindern. Präv Gesundheitsf.

[17] Soellner R, Huber S, Reder M (2014). The concept of ehealth literacy and its measurement. German translation of the eHEALS. J Media Psychol.

[18] Ghaddar SF, Valerio MA, Garcia CM, Hansen L (2012). Adolescent health literacy: the importance of credible sources for online health information. J Sch Health.

[19] Korkmaz Aslan G, Kartal A, Turan T, Taşdemir Yiğitoğlu G, Kocakabak C (2021). Association of electronic health literacy with health-promoting behaviours in adolescents. Int J Nurs Pract.

[20] Levin-Zamir D, Lemish D, Gofin R (2011). Media health literacy (MHL): development and measurement of the concept among adolescents. Health Educ Res.

[21] Lam LT, Lam MKP (2015). Competency of health information acquisition and intention for active health behaviour in children. Int Arch Med.

[22] Schienkiewitz A, Brettschneider A-K, Damerow S, Schaffrath Rosario A (2018). Übergewicht und Adipositas im Kindes- und Jugendalter in Deutschland – Querschnittergebnisse aus KiGGS Welle und Trends. J Health Monit.

[23] van der Vaart R, Drossaert C (2017). Development of the digital health literacy instrument: measuring a broad spectrum of health 1.0 and health 2.0 skills. J Med Internet Res.

[24] Dadaczynski K, Okan O, Messer M, Leung A, Rosário R, Darlington E, Rathmann K (2021). Digital health literacy and web-based information-seeking behaviors of university students in Germany during the COVID-19 pandemic: cross-sectional survey study. J Med Internet Res.

[25] Bucksch J, Häußler A, Schneider K, Finne E, Schmidt K (2020). Bewegungs- und Ernährungsverhalten von älteren Kindern und Jugendlichen in Deutschland – Querschnittergebnisse der HBSC-Studie 2017/18 und Trends. J Health Monit.

[26] Inchley J, Currie D, Cosma A (2018). Health behaviour in school-aged children (HBSC) study protocol: background, methodology and mandatory items for the 2017/18 survey.

[27] Hoebel J, Müters S, Kuntz B, Lange C, Lampert T (2015). Messung des subjektiven sozialen Status in der Gesundheitsforschung mit einer deutschen Version der MacArthur Scale. Bundesgesundheitsblatt Gesundheitsforschung Gesundheitsschutz.

[28] Zarocostas J (2020). How to fight an infodemic. Lancet.

[29] Paakkari L, Okan O (2020). COVID-19: health literacy is an underestimated problem. Lancet Public Health.

[30] Vodafone Stiftung Deutschland (2020) Die Jugend in der Infodemie. Eine repräsentative Befragung zum Umgang junger Menschen in Deutschland mit Falschnachrichten während der Coronakrise. https://bit.ly/3djUsN6. Zugegriffen: 4. Dez. 2021

[31] Freeman JL, Caldwell PHY, Bennett PA, Scott KM (2018). How adolescents search for and appraise online health information: a systematic review. J Pediatr.

[32] Sligo FX, Jameson AM (2000). The knowledge-behavior gap in use of health information. J Am Soc Inf Sci Technol.

[33] Rhodes RE, de Bruijn G-J (2013). How big is the physical activity intention-behaviour gap? A meta-analysis using the action control framework. Br J Health Psychol.

[34] Fleary SA, Joseph P (2020). Adolescents’ health literacy and decision-making: a qualitative study. am j health behav.

[35] Quenzel G, Schaeffer D, Messer M, Vogt D (2015). Gesundheitskompetenz bildungsferner Jugendlicher. Einflussfaktoren und Folgen. Bundesgesundheitsblatt Gesundheitsforschung Gesundheitsschutz.

[36] Bollweg TM, Okan O, Fretian A, Janner C, Schulenkorf T, Pinheiro P, Bauer U (2021). Dimensionen der Gesundheitskompetenz von Viertklässler*innen. Ergebnisse einer explorativen Querschnittstudie in Nordrhein-Westfalen. Präv Gesundheitsf.

[37] Chang L-C (2011). Health literacy, self-reported status and health promoting behaviours for adolescents in Taiwan. J Clin Nurs.

[38] Zhang F, Or PPL, Chung JWY (2021). How different health literacy dimensions influences health and well-being among men and women: the mediating role of health behaviours. Health Expect.

[39] Walters R, Leslie SJ, Polson R, Cusack T, Gorely T (2020). Establishing the efficacy of interventions to improve health literacy and health behaviours: a systematic review. BMC Public Health.

[40] Park E, Kwon M (2021). Testing the digital health literacy instrument for adolescents: cognitive interviews. J Med Internet Res.

[41] Altman DG, Royston P (2006). The cost of dichotomising continuous variables. BMJ.

[42] Schulenkorf T, Krah V, Dadaczynski K, Okan O (2021). Addressing health literacy in schools in Germany: concept analysis of the mandatory digital and media literacy school curriculum. Front Public Health.

